# Abnormalities in hubs location and nodes centrality predict cognitive slowing and increased performance variability in first-episode schizophrenia patients

**DOI:** 10.1038/s41598-019-46111-0

**Published:** 2019-07-03

**Authors:** Paweł Krukow, Kamil Jonak, Robert Karpiński, Hanna Karakuła-Juchnowicz

**Affiliations:** 1 Department of Clinical Neuropsychiatry, Medical University of Lublin, Lublin, Poland; 2 Department of Biomedical Engineering, Lublin University of Technology, Lublin, Poland; 3 Chair and I Clinic of Psychiatry, Psychotherapy and Early Intervention, Medical University of Lublin, Lublin, Poland; 4 Department of Machine Design and Mechatronics, Faculty of Mechanical Engineering, Lublin University of Technology, Lublin, Poland

**Keywords:** Attention, Schizophrenia

## Abstract

Introducing the Minimum Spanning Tree (MST) algorithms to neural networks science eliminated the problem of arbitrary setting of the threshold for connectivity strength. Despite these advantages, MST has been rarely used to study network abnormalities in schizophrenia. An MST graph mapping a network structure is its simplification, therefore, it is important to verify whether the reconfigured network is significantly related to the behavioural dimensions of the clinical picture of schizophrenia. 35 first-episode schizophrenia patients and 35 matched healthy controls underwent an assessment of information processing speed, cognitive inter-trial variability modelled with ex-Gaussian distributional analysis of reaction times and resting-state EEG recordings to obtain frequency-specific functional connectivity matrices from which MST graphs were computed. The patients’ network had a more random structure and star-like arrangement with overloaded hubs positioned more posteriorly than it was in the case of the control group. Deficient processing speed in the group of patients was predicted by increased maximal betweenness centrality in beta and gamma bands, while decreased consistency in cognitive processing was predicted by the betweenness centrality of posterior nodes in the gamma band, together with duration of illness. The betweenness centrality of posterior nodes in the gamma band was also significantly correlated with positive psychotic symptoms in the clinical group.

## Introduction

Schizophrenia is a progressive disease characterized by complex psychopathology and cognitive dysfunctions impeding the abilities of independent living^1^. A significant part of modern studies concerning the neural underpinnings of the disorder are focused on recognizing the patterns of aberrant brain integration. It resulted from the assumption that schizophrenia (SZ) might be understood as a pathological state of disconnection within the neural system^2,3^. The disconnection or neural miswiring^4^ refers to a pathology in the coordination of brain elements from the level of cellular signalling to aberrant axonal connections between various neuroanatomical structures^5^. Much of the evidence for desynchronized neural activity in SZ comes from research using resting-state functional magnetic resonance methods (r-s fMRI), aimed at finding enhanced or reduced functional connectivity between numerous brain areas and networks^6,7^. Functional connectivity (FC) might be defined as the statistical association between spatially distributed neurophysiological time series^8^. Despite a substantial increase in the scope of knowledge regarding abnormal FC in SZ individuals^9,10^, an infinite number of possible connections between different brain regions makes it difficult to assess the condition of the network in a global dimension. To circumvent this problem, a graph theorem has been implemented to evaluate the complex whole-brain functional and structural networks^11–13^. Moreover, implementation of graph theory makes possible the transition from investigating associations between a given number of structures to interpreting overall connectivity configuration^14^.

Previous studies of neural networks organization in schizophrenia confirmed various types of pathologies, however, a substantial inconsistency of detailed results has been noted. The inconsistency was mainly due to the application of various solutions regarding networks and connectivity metrics and dissimilar modalities of neuroimaging technics^15^. Kambeitz with co-workers^16^ conducted a meta-analysis of the whole-brain network architecture to verify whether the integrity of brain functioning is globally disrupted in schizophrenia. The authors selected eight fMRI and five EEG studies. In general, the results based on three r-s fMRI studies of SZ samples showed significant decrease of the small-worldness, a metric indicative of appropriate balance between local segregation and global integration of the network. What is more, patients also had a significantly reduced clustering coefficient, showing the extent of regional density or a tendency toward the development of cliquishness within the network, and diminished local efficiency indexing the fault tolerance of the network. Two r-s EEG studies demonstrated non-significant decrease in minimal path-length, a measure associated with integration of the network. Another r-s EEG study also confirmed the decreased clustering coefficient in the SZ group. Analytical results showed reduced local organization of the patients’ network, but also marked that the main limitation of the included research concerned arbitrariness in setting a threshold for connectivity strength, which could be a major reason for the heterogeneity of the results. It is also worth noting that Kambeitz *et al*.^16^ were able to find only two r-s EEG studies^17,18^ which significantly differentiated the global network’s organization of SZ patients and controls.

Apart from the assessment of the overall neural activity arrangement, the research also concerned the evaluation of networks at the regional level, in order to identify neural circuits differentiating SZ patients and controls in functional connectivity strength. Alamian and co-workers^15^ have attempted to indicate results typical for patients across various neuroimaging modalities (MEG, EEG, fMRI), and suggested that FC within the frontal cortex can be assumed as definitively altered in SZ, regardless of the neuroimaging method used. For example, Zhu *et al*.^19^ showed that SZ patients exhibited increased nodal centrality in central and posterior regions and decreased centrality in anterior and subcortical structures compared with controls. Additionally, almost all graph theory-driven research in SZ refers to the number and the location of the so called central hubs. Hubs are highly connected nodes, characterized by strategic connectivity facilitating the integration of the network^20^. In healthy individuals, central hubs have been located mainly, though not exclusively, within the anterior areas including the prefrontal cortex^21,22^ and, according to Rubinov and Bullmore^23^, various abnormalities regarding the anterior hubs, with fewer hubs located in the frontal cortex and the emergence of more non-frontal hubs, are typical features of SZ patients’ networks^24,25^.

Compared to various types of imaging methods based on magnetic resonance, electro- and magnetoencephalography (MEG) enables the identification of altered oscillatory synchronization occurring in millisecond-range resolution, grasped in several frequencies, including the fastest ones, e.g. above 30 Hz, which are beyond the temporal range for methods based on MRI^26^. Therefore, new neurophysiological studies are needed, with an application of graph-theory algorithms not burdened with computational issues such as the arbitrary setting of the threshold for the connectivity strength, and including a regional analysis of neural networks.

**Figure 1:**
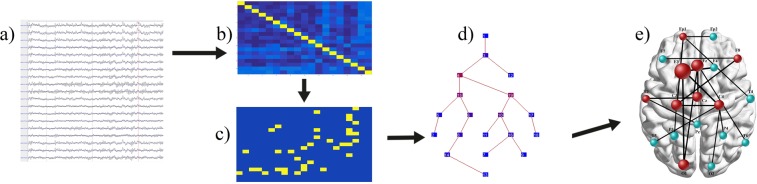
Schematic illustration of MST-networks formation from the resting-state EEG recordings: (**a**) selection of thirty EEG artifacts-free epochs, each containing 4096 samples (approximately 8 seconds per epoch) from each participants, (**b**) computation of the functional connectivity matrices based on phase lag index (PLI) for every selected EEG epochs for each possible electrodes pairs, in every frequency band, (**c**) minimum spanning tree (MST) matrix calculation, based on previously obtained PLI matrices for all frequencies, (**d**) the MST graph reconstruction, being an output from the Brainwave software, (**e**) illustrative reconstruction of topological distribution of hubs and leaf nodes posted on a simplified cortical surface, created from averaged MST matrix in a given frequency band. Network was reconstructed keeping the rule of generating loop-less connections between the nodes. Analyses and computations depicted in points (**a**–**d**) were performed for each individual participant, while the topological network reconstruction (point **e**) has been prepared for groups, to show a qualitative model of networks with the approximate neuronal location of hubs, leaf nodes and connections.

One of the methods eliminating the thresholding problem is the application of the Minimum Spanning Tree (MST). MST enables the reconstruction of an acyclic, loop-less subgraph of the original undirected, weighted graph in which all nodes are connected^27,28^. The output of the MST analysis contains only a subset of the strongest connections representing a stable network’s core and allows an unbiased comparison of networks differing in its density (Fig. 1). To date, many studies have been carried out using MST to reconstruct the global neural networks in such clinical conditions as, for example, epilepsy^29,30^, stuttering^31^, Alzheimer’s disease^32^ and in brain maturation research^33^. These studies confirmed the usefulness of the MST algorithms to identify network irregularities in the mentioned populations. Moreover, van Diessen with co-workers^30^ proved that MST metrics differentiated epileptic patients better from controls in terms of their network’s organization than the conventional graph-theory indicators. As it was demonstrated in the study of Engels *et al*.^32^, MST might be also applied to regional analysis of the network, by establishing the betweenness centrality (BC, a measure of the hub-status) for each node (EEG electrodes) and then averaging BC results for nodes assigned to anterior, central and posterior regions. This method yielded valuable results since it was shown that BC of central and anterior regions increased with accumulating disease severity associated with progressive neurodegeneration of more posterior areas.

In SZ studies, MST has been implemented to a small extent so far. Van Dellen *et al*.^34^ assessed structural brain networks with an application of MST to DTI data in groups of subclinical and clinical psychosis. Authors corroborated decreased centrality of parietal and language areas in both clinical samples. Another MST-based^35^ study showed higher connection entropy in gamma frequency in the SZ group than in the controls’ group, yet, MST metrics such as diameter, leaf  fraction or BC were not compared between the included groups. Jonak with co-workers^36^ conducted an r-s EEG study to contrast MST-derived networks indices in first-episode and multi-episode SZ patients. They demonstrated that the network of multi-episode patients was more centralized as compared to the first-episode sample, however, a group of healthy controls did not participate in this analysis.

Taking the presented data into account, it seems reasonable to broaden the existing findings regarding the global organization of the network in schizophrenia with a study based on the r-s EEG recordings in which the network will be reconstructed using well-validated MST metrics in SZ patients and healthy controls. In addition, to our knowledge, no study has yet been conducted to verify whether the expected abnormalities in SZ patients’ EEG-derived networks, measured by MST, are significant predictors of cognitive deficits and psychopathological symptoms typical for schizophrenia. This is of special importance, considering that although MST provides well-defined subnetworks, yet, to some extent generates only a simplified model of the original network, without connections forming loops^27^. Consequently, it should be validated, whether the neural network reconstructed with the use of MST adequately reflects the abnormal organization of neuronal activity of SZ patients, and is associated with behavioural indicators.

Therefore, the main goal of this study was to evaluate the architecture of neural networks in a group of SZ patients and controls based on r-s EEG recordings, with an application of such global MST metrics as diameter, leaf fraction and betweenness centrality. Additionally, we also intend to compare the groups in terms of regional characteristics, similarly as in Engels *et al*.^32^ study. Basing on the previously described results, we expect that the patient network will contain more hubs in the posterior cortical areas compared with controls. Another aim was to investigate which of the network features will significantly predict the expected cognitive dysfunctions such as cognitive slowing and increased variability of information processing.

We chose these two cognitive dimensions for two reasons: earlier studies demonstrated that impaired processing speed significantly contributes to differences between patients, their siblings and healthy individuals regarding any other cognitive dimensions^37–39^. Because cognitive speed in SZ patients is related to white matter integrity^40,41^ and strength of long-range synchronization^42^, we assumed that its disruption should be associated with global network properties, such as centrality or assortativity. Secondly, much of the research carried out so far has confirmed universally heightened cognitive variability in SZ subjects and their healthy siblings^43–45^. According to fundamental ideas of Fox and Raichle^46,47^ regarding the nature of resting-state brain dynamics, behavioural inter-trial variability is considered as the genuine manifestation of spontaneous fluctuations within the intrinsic, distributed cortical systems. Therefore, we postulate that the network abnormalities examined in the off-task condition should also be associated with increased cognitive variability in SZ. Specifically, increased intra-individual variability should be associated with altered centrality of anterior/frontal region, considering the neuropsychological models of cognitive inconsistency emphasizing its correlations with dysexecutive syndrome^48,49^.

## Results

### Clinical and cognitive data

Table 1 presents demographic, clinical and cognitive characteristics of the studied groups. Samples did not differ in terms of age (SZ = 21.14; HC = 21.54), gender (SZ = 48.% male; HC = 42.8% male), years of education (SZ = 13.85; HC = 14.20) and premorbid intelligence (SZ = 107.60; HC = 107.82). In SZ group, the duration of illness was about 1 year, and the average time of untreated psychosis lasted no more than half a year. All patients were treated with atypical antipsychotics: 65.62% of patients with Olanzapine, 22.85% were taking Risperidone and 11.53% Aripiprazole.

**Table 1:** Demographic, clinical and cognitive characteristics of the studied groups

Characteristics	SZ (n = 35) M (SD)	HC (n = 35) M (SD)	Statistic value
Age (years)	21.14 (2.95)	21.54 (0.70)	F (1, 68) = 0.60, *p* = 0.438, η_p_^2^ = 0.008
Male/female	17/18	15/20	χ^2^ = 0.23, df = 1, *p* = 0.631
Education (years)	13.85 (1.86)	14.20 (1.36)	F (1, 68) = 0.76, *p* = 0.383, η_p_^2^ = 0.011
Premorbid IQ	107.60 (6.66)	107.82 (7.52)	F (1, 68) = 0.06, *p* = 0.893, η_p_^2^ = 0.001
Duration of illness (months)	12.31 (5.65)	NA	NA
Duration of untreated psychosis (months)	4.85 (4.79)	NA	NA
PANSS positive	14.34 (4.73)	NA	NA
PANSS negative	19.25 (6.14)	NA	NA
PANSS general	34.87 (6.12)	NA	NA
PANSS total	68.34 (10.53)	NA	NA
Risperidone equivalents	4.37 (1.48)	NA	NA
**Cognitive speed:**			
Total number of processed stimuli	55.20 (13.16)	74.20 (7.24)	F(1, 65) = 54.60, ***p*** **<** **0**.**0001**, **η**_**p**_^**2**^ **=** **0**.**456**^b^
Total number of errors	3.11 (3.17)	1.85 (2.39)	F(1, 65) = 3.51, *p* = 0.065, η_p_^2^ = 0.051^b^
RT_mean_	1685.76^a^ (414.15)	1212.39 (121.16)	F(1, 65) = 41.74, ***p*** **<** **0**.**0001**, **η**_**p**_^**2**^ **=** **0**.**381**^b^
iSD	967.09^a^ (599.86)	602.55 (327.48)	F(1, 65) = 11.19, ***p*** **=** **0**.**001**, **η**_**p**_^**2**^ **=** **0**.**146**^**b**^
**Ex-Gaussian indicators:**			
*μ*	990.44^a^ (170.91)	765.19 (68.91)	F(1, 65) = 51.52, ***p*** **<** **0**.**0001**, **η**_**p**_^**2**^ **=** **0**.**442**^b^
*σ*	78.31^a^ (63.78)	90.81 (62.52)	F(1, 65) = 1.37, *p* = 0.244, η_p_^2^ = 0.019^b^
*τ*	673.93^a^ (333.09)	455.23 (143.84)	F(1, 65) = 13.65, ***p*** **=** **0**.**0004**, **η**_**p**_^**2**^ **=** **0**.**173**^b^

Notes: SZ = first-episode schizophrenia patients, HC = healthy controls, PANSS = Positive and Negative Syndrome Scale, assessed during remission phase, NA = not applicable, RT_mean_ = mean reaction time, iSD = individual standard deviation.

^a^all data given in milliseconds, ^b^ANCOVA with age, education and premorbid IQ as controlled covariates.

Bold font indicates statistically significant effects, including Bonferroni correction for multiple testing.

As for the cognitive results concerning the processing speed and variability, SZ patients had a significantly worse outcome comparing with controls. Specifically, they processed less stimuli in a given amount of time and their mean RT was longer. Moreover, the application of RTs ex-Gaussian modelling indicated significantly longer average RT (*μ* parameter) and heightened variability of processing referring to the exponential part of the distribution with the most prolonged RTs (*τ* parameter, Table 1). No significant group difference emerged concerning the number of errors committed in the cognitive speed task. In the SZ sample, there was no significant correlation between *μ* and *τ* (r = 0.03, *p* = 0.864), whereas it emerged in HC group: r = −0.53, *p* = 0.001. Further, in SZ group, the total number of stimuli processed in DST was significantly predicted by *τ* (β = −0.79) and *μ* parameters (β = −0.54): R^2^ = 0.97, F(6, 28) = 235.44, *p* < 0.0001. An indicator of cognitive inconsistency (*τ*) correlated with the subscale of negative symptoms from PANSS administrated in remission phase (R = 0.48, *p* = 0.002).

### Functional connectivity and networks organization in the studied groups

**Figure 2:**
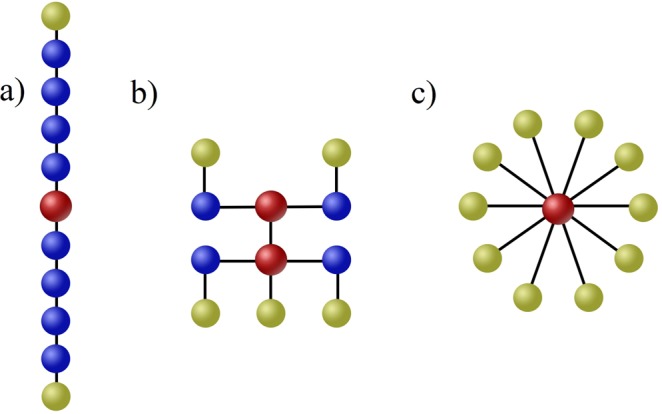
Delineative representation of minimum spanning trees with reference to the network types. (**a**) shows a path-like configuration, in which two end nodes are the leafs (light green) of the tree. Such model network has low leaf fraction, high diameter and low betweenness centrality. (**b**) presents a diagram of a well-balanced network containing five nodes and two hubs creating a “rich club”, constituted by two directly connected nodes with increased centrality. Relatively small amount of leaf nodes prevents the hubs from overloading. (**c**) is an example of a star-like tree, having a central, overloaded hub (red) connected with all nodes. This network is characterized by high leaf fraction, low diameter and high betweenness centrality. In an extreme form of a path-like network segregation processes prevail, because it takes a lot of steps to transfer information from the initial to the last node, while an extreme star-like network is dominated by integration processes because information flow from all nodes reaches one central hub and overload it.

**Figure 3:**
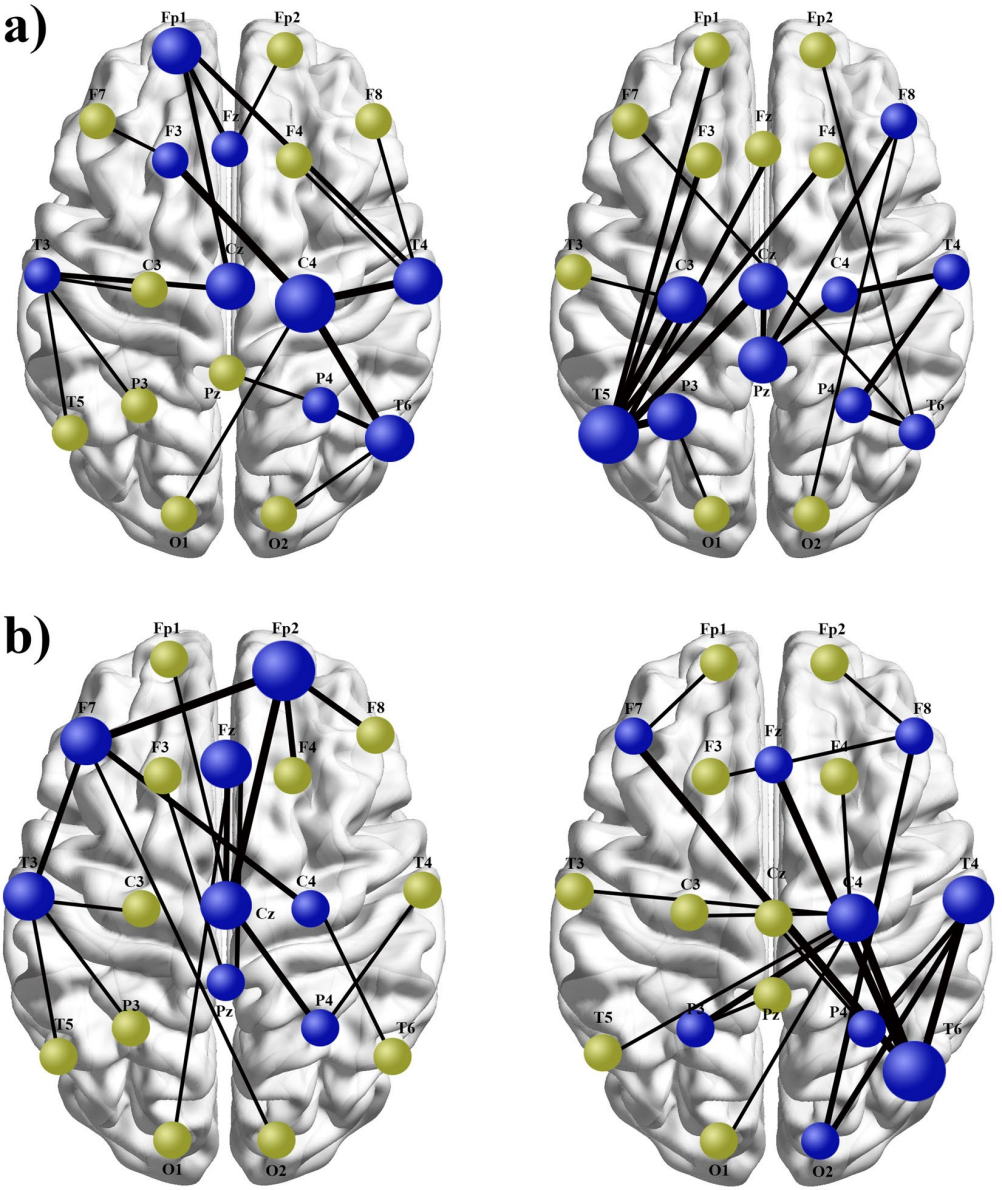
An illustrative comparison of the MST topologies differing with respect to: increased and decreased maximal betweenness centrality (**a**), and comparison of regionally diversified topologies with regard to betweenness centrality of nodes located in anterior *versus* posterior areas (**b**). Blue nodes are hubs, while light green nodes are leafs. The thickness of the lines corresponds to functional connectivity values based on the phase lag index algorithm. (**a**). Topography on the right side contains severely overloaded central hub (T_5_ electrode) positioned in the left temporal lobe, directly connected to seven other nodes. Compared with this substantially centralized topology, network organization showed on the left does not lead to hubs overloading, due to more balanced and dispersed distribution of connections between individual nodes. (**b**). The organization shown on the right contains more hubs located in the posterior part of cortical surface, especially in the right temporal and parietal lobes. The central hub, with the maximal BC in the entire network is located in the right temporal lobe (electrode T_6_). The topology displayed on the left contains more hubs in the anterior area and the central hub positioned within the right prefrontal cortex (electrode F_p_2). Shown topologies were reconstructed according to pipeline presented in Fig. 1., on the basis of MST results obtained from our SZ patients (on the right) and healthy controls (on the left) in beta (**a**) and gamma (**b**) frequencies (see Supplementary Information: Figs S1 and S2. for row MST graph of both groups). Described between-groups differences were confirmed in statistical comparisons regarding BC_max_ in beta band and regional betweenness centrality in gamma frequency (Table 2).

MST analysis revealed significant group effects for the majority of the studied frequencies (Table 2). In delta band, SZ patients had a lower diameter and higher leaf fraction relative to the control group, indicating that their network contained a higher amount of the shortest paths and increased number of nodes with only one connection. Degree correlation in patients (R = −0.45) was higher than in the control group (R = −0.31) confirming increased network disassortativity in the SZ group. Additionally, a regional analysis showed higher BC for the posterior region in SZ, compared to the control group. Between-groups effects in theta band concerned stronger oscillatory synchronization (PLI) in SZ patients compared to the control group. The reverse result in terms of synchronization strength was determined in the lower alpha frequency range. No intergroup differences were recorded in the upper alpha frequency. Similarly to the delta band, the network of SZ patients in the beta frequency was characterized by the increased number of shortest paths (diameter) and lower global centrality. Moreover, in the SZ group, the network had a stronger tendency to develop individual hubs with substantially increased maximal betweenness centrality (BC_max_). Significant between-group effects concerning functional connectivity and MST metrics were also identified in gamma frequency. Generally, the SZ sample had a shorter largest distance between any two nodes (diameter), accompanied with higher leaf fraction. The patients’ network was substantially more centralized as indicated by more hubs with maximum centrality (BC_max_) positioned mainly in the posterior region as compared to the control group (see Figs 2 and 3). Within the theta and lower alpha bands, the PLI parameters were not correlated with the MST indices that significantly differentiated the studied groups.

**Table 2:** PLI and MST metrics significantly differentiated the studied groups and correlations between these indicators and ex-Gaussian parameters *μ* and *τ* in first-episode schizophrenia group (SZ).

PLI and MST results	SZ *versus* HC	η_p_^2^	Correlations between MST and ex-Gaussian parameters	
			*μ*	*τ*
***Delta***				
PLI	—	0.007		
Diameter	**↓**	**0**.**100**	−0.04	0.14
Leaf fraction	**↑**	**0**.**131**	−0.11	−0.22
R	**↑**	**0**.**139**	0.17	−0.28
BC_max_	—	0.025		
BC global	—	0.042		
BC anterior	—	0.001		
BC posterior	↑	**0**.**128**	0.06	−0.05
***Theta***				
PLI	↑	**0**.**321**	0.15	0.28
Diameter	—	0.021		
Leaf fraction	—	0.076		
R	—	0.067		
BC_max_	—	0.018		
BC global	—	0.007		
BC anterior	—	0.035		
BC posterior	—	0.045		
***Lower alpha***				
PLI	↓	**0**.**136**	−0.26	0.28
Diameter	—	0.001		
Leaf fraction	—	0.002		
R	—	0.003		
BC_max_	—	0.001		
BC global	—	0.001		
BC anterior	—	0.007		
BC posterior	—	0.073		
***Upper alpha***				
PLI	—	0.006		
Diameter	—	0.015		
Leaf fraction	—	0.001		
R	—	0.026		
BC_max_	—	0.023		
BC global	—	0.001		
BC anterior	—	0.007		
BC posterior	—	0.074		
***Beta***				
PLI	—	0.052		
Diameter	↓	**0**.**093**	−0.08	0.013
Leaf fraction	—	0.045		
R	—	0.003		
BC_max_	↑	**0**.**122**	**0**.45*	−0.19
BC global	↓	**0**.**100**	−0.39	0.21
BC anterior	—	0.001		
BC posterior	—	0.048		
***Gamma***				
PLI	—	0.076		
Diameter	↓	**0**.**234**	−**0**.45*	0.17
Leaf fraction	↑	**0**.**244**	0.23	0.21
R	—	0.035		
BC_max_	↑	**0**.**205**	**0**.47*	0.29
BC global	—	0.019		
BC anterior	—	0.006		
BC posterior	↑	**0**.**235**	0.01	**0**.72**

Note. Bold *η*_*p*_^2^ represents significant effect sizes, ↑ result significantly (p < 0.01) higher in SZ group (n = 35, HC n = 35), ↓ result significantly (p < 0.01) lower in SZ group, -n.s. Correlations significance *p < 0.01, **p < 0.001

Bold font indicates statistically significant effects, including Bonferroni correction for multiple testing

### Correlations between MST metrics, demographic and clinical variables in the SZ group

There were no significant correlations between the network parameters differentiating the studied groups and age, education, premorbid IQ, duration of disease, DUP and risperidone equivalent in the SZ sample (*p* > 0.05). Only one significant correlation was found between MST metrics and PANSS scale, which concerned the relationship between the subscale of positive symptoms and BC_posterior_ in gamma band (R = 0.51, p = 0.002).

### Associations between networks’ characteristics and ex-Gaussian indicators of cognitive speed and variability in the SZ group

As presented in Table 2, in the SZ group *μ* parameter significantly correlated with BC_max_ (r = 0.45, *p* = 0.006) in beta frequency and with BC_max_ (r = 0.47, *p* = 0.004) and diameter (r = −0.45, *p* = 0.006) in gamma frequency. The exponential part of the distribution with the most prolonged RTs (*τ*) correlated with betweenness centrality of posterior nodes in gamma (r = 0.72, *p* < 0.001) frequency.

Because the ex-Gaussian parameters’ distribution, taking into account the Kolmogorov-Smirnov test results (*p* > 0.1), did not deviate from normal, regression analysis was performed to verify whether the network characteristics analyzed with MST prove to be significant predictors of information processing speed and variability, also when such variables as the duration of illness, DUP and the risperidon equivalent were controlled.

**Figure 4:**
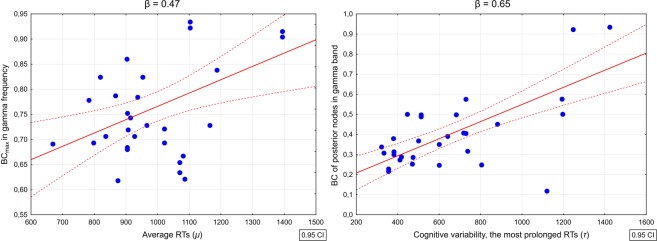
Correlation plots showing relationships between selected neural networks metrics and the ex-Gaussian parameters characterizing the distribution of reaction times (RTs) derived from the cognitive speed task performed by patients with first-episode schizophrenia. Left panel represents correlations between maximal betweenness centrality in gamma frequency and the average RTs (*μ* parameter), while the right panel displays the correlation between the centrality of nodes localized in the posterior part of the cortex and the most prolonged RTs being an indicator of processing inconsistency (*τ* parameter). In both cases, depicted associations were significant also after controlling for duration of untreated psychosis, duration of illness and risperidone equivalent.

Multivariate regression analysis revealed that average RTs (*μ*) was significantly predicted by BC_max_ in gamma frequency (β = 0.47), BC_max_ in beta frequency (β = 0.45) and DUP (β = 0.32): R^2^ = 0.58, F(7, 27) = 9.12, *p* < 0.0001, also after controlling for duration of illness and risperidone equivalent. Significant predictors were not correlated with each other (*p* > 0.05). The betweenness centrality of posterior nodes in gamma band (β = 0.65), together with duration of illness (β = 0.26) turn out to significantly predict the cognitive variability (*τ*) in SZ group: R^2^ = 0.62, F(5, 29) = 9.47, *p* < 0.0001, also when the rest of the controlled clinical variables were included (Fig. 4).

## Discussion

The main goal of this study was to verify whether the neural network reconstructed using the MST algorithm on the basis of resting-state EEG recording would significantly differentiate SZ patients from healthy controls. The second goal was establishing whether the network parameters differentiating the indicated groups would be associated with behavioural variables such as cognitive deficits and psychopathological symptoms. Generally, the obtained results partially confirmed the formulated hypotheses.

Our analysis of the MST output showed that patients’ network, as compared to controls, was substantially less efficiently organized, with its arrangement resembling a star-like composition. Moreover, the networks’ disassortativity was also higher in the SZ group, however, this concerned only the delta frequency. A star-like organization in an exaggerated form leads to deficient intra-network communication, due to the overloading of the prominent hubs, impeding the effective exchange of information within the network^29,50^. In the beta band, increased maximal betweenness centrality (BC_max_) was accompanied by decreased mean betweenness centrality, which additionally suggests an imbalance in hubs strength. Importantly, such a relationship was not observed among the healthy controls. A heightened network randomness in the SZ group refers to a network construction with central hubs directly attached to low degree nodes, preventing the possibility to create the so called “rich clubs”^27,51^. This means that, if a more integrated, centralized network is concomitant with intensified disassortativity, then it does not provide the optimal level of performance, as in the case of our SZ patients.

Tewarie with co-workers^52^ have attempted to translate MST network-specific indices to the terminology of conventional graph theoretical measures, especially average path length and small-worldness. The authors noted that such a translation is possible, although due to the specifics of the Kruskal’s algorithm, eliminating any loops from the final MST topology, there is probably no direct equivalent of clustering coefficient. However, considering global MST results in our groups, it can be concluded that the average path length in the SZ group was significantly shorter than in the group of controls^52^. Considering that SZ patients’ network might be defined as having a star-like organization, it seems, that it is significantly different from a small-world network, in which local specialization and global integration are well-balanced. A star-like network with strong, but overloaded hubs, might be rather classified as scale-free topological organization constructed in accordance with the power law degree principle^53^. Although scale-free configurations were observed in researches on brain networks^20^, a significant shift towards over-centralization of the network in SZ group suggest a violation of the small-worldness rule in patients’ topologies.

Regional network analysis revealed that SZ participants had significantly more hubs located in posterior areas as compared to controls. This evaluation seems to confirm the previously discussed observations of other authors^24,25^ who found the presence of a larger number of non-frontal hubs in SZ patients.

Generally, the results of this study, providing additional evidence of abnormal network organization in SZ patients, are relatively consistent with the previous findings. Other authors also demonstrated significantly weakened centrality within the frontal regions in SZ, interpreted as a reduced role of the frontal cortex in the network global integration^54^. Rubinov *et al*.^18^ revealed decreased overall hubs mean centrality and shorter path length. Additionally, Jalili and Knyazeva^55^, utilizing resting-state EEG also reported shortening of the path length and decreased nodal strength of the anterior hubs in schizophrenia. Further, studies using structural imaging methods (MRI, DTI) confirmed the reduction of frontal hubs strength as well as the appearance of non-frontal hubs^56,57^.

As expected, MST metrics which differentiated the studied groups were also associated with behavioural characteristics of SZ patients. A relatively surprising finding was the establishing of only one significant correlation between an abnormal feature of network configuration and the psychopathology of schizophrenia. Specifically, it concerned the association between the subscale of positive psychotic symptoms and the centrality of the posteriorly located nodes in the gamma frequency. Schoen *et al*.^35^ noticed that heightened connection entropy in gamma band correlated with a pathologically high PANNS outcome collected before starting the antipsychotic treatment, but after treatment and having achieved a significant clinical improvement, these correlations were no longer statistically significant. Considering the mentioned results, Schoen with co-workers^35^ suggested that abnormal connectivity formation in patients is correlated with the diagnosis of schizophrenia, but not with symptomatology changing in response to pharmacological treatment.

In addition to the analysis of irregularities in the organization of neural networks in schizophrenia, we also demonstrated that the overall poor score in the cognitive speed test in SZ group was predicted by two unrelated variables: the increased average response times (RTs) and processing variability in a form of the most elongated RTs. The elongated average RTs were predicted by increased BC_max_ in beta and gamma bands, together with the duration of untreated psychosis. The positive correlations between BC_max_ in higher frequencies and the most typical patients’ RTs mean that the response time extended with increasing centralization of the strongest nodes in the network. A topological organization of a network not secured against the overload of the central hubs suffers from a serious imbalance between integration and selection in information processing^50^. It seems, therefore, that the inefficient selectivity in networks performance might limit the ability of fast cognitive performance. The ex-Gaussian indicator of cognitive variability covering the most elongated RTs (*τ*), which explained even a larger scope of cognitive speed variance than the average RTs, was predicted by betweenness centrality of posterior nodes in gamma band and the duration of illness in the SZ sample. As marked earlier, schizophrenia is associated with a greater density of highly centralized nodes in the posterior area of the cerebral cortex. The backward-shifting of hubs proved to be a significant predictor of the increasing inconsistency during the cognitive speed task in the SZ group. Associations between failures of attentional-executive control over processing speed task and more posteriorly located hubs in our SZ group might be interpreted with reference to the previous findings^58^ confirming that the strength of anti-correlation between the ‘task-negative’ (e.g. default-mode network) and ‘task-positive’ networks, explained a significant part of cognitive instability variation in healthy participants. Fassbander *et al*.^59^ also found that reduced activity of the dorsolateral prefrontal cortex in schizophrenia was accompanied by very long RTs during the Stroop task. This effect was interpreted as an evidence of insufficient recruitment of cognitive control networks during trials with prolonged RTs. It should be noted however that the comparison of the groups included in our study did not show that SZ patients had statistically significant lower betweenness centrality across the anterior/frontal area. Therefore, we cannot fully explain our findings with reference to the indicated relationships between ‘de-frontalization’ of the network and the failure of attentional-executive control, as was suggested in the study of Fassbander *et al*.^59^.

Despite several strengths of our investigation, notably, demonstrating that the aberrant properties of the neural networks arrangement reconstructed with the use of MST algorithms in schizophrenia have significant behavioural effects, this research has some limitations which should be addressed. First, our study was correlational in its nature, which limited the possibility of experimental manipulation of the assessed variables. Secondly, fluctuations in cognitive processing and neural activity were not induced by changes of the task requirements. However, our aim was to register the spontaneous variation in information processing as a function of assumed networks abnormality. The yielded results seem to confirm the validity of the applied methodology since in the present study the specific neural networks’ features in the SZ group explained a substantial range of variance in cognitive performance. Thirdly, to determine the organization of the neural activity, resting-state EEG was used, with a relatively small amount of electrodes, what could have restricted the precision of nodes location. Also, due to the application of PLI to measure functional connectivity, this location covers only the cortical surface. Nevertheless, since we wanted to characterize the global network organization and its relationship to psychopathology and cognitive impairment, such a procedure seems to be justified, as shown by our and previous studies^30,31^.

In the end, it should be accentuated that the results of our investigation confirm the pivotal role of deficient gamma synchronization in schizophrenia. Within this frequency the largest number of MST metrics differentiated the groups of SZ patients from healthy controls, these metrics most often turned out to be significantly related to clinical symptoms and analyzed neuropsychological deficits. Extensive reviews and empirical research unequivocally confirm the critical engagement of gamma oscillations in the neural underpinning of schizophrenia^60,61^, its relations with a psychotic onset and disturbed neurochemical processes, especially glutamatergic neurotransmission via the NMDA (N-methyl-D-aspartate) receptors^62–64^. Therefore future studies focused on establishing links between electrophysiological activity and schizophrenia should always refer to high-frequency neural dynamics.

## Methods

### Participants

All participants met the following inclusion criteria: age between 18 and 25 years old, right-handedness and at least 12 years of education, while the exclusion criteria concerned taking benzodiazepines, anticholinergic agents and mood stabilizers up to three months before the assessment. Ultimately, a group of 35 inpatients diagnosed as first-episode schizophrenia (SZ) according to DSM-5 classification (Structured Clinical Interview for DSM-5, SCI) and 35 healthy controls (HC) participated in this study. Patients assigned to the research group were recruited from the Department and Clinic of Psychiatry, Psychotherapy and Early Intervention of the Medical University of Lublin, Poland. The HC group was recruited from the local community after the clinical group was completed to guarantee the demographic matching of individuals from both samples. In addition to the mentioned general inclusion and exclusion criteria, healthy controls had to meet additional conditions such as: lack of positive family history of psychotic and affective diseases disclosed, no personal history of Axis I diagnoses as established by the SCID for DSM-5 Axis I Disorders, neurological injuries or brain disease revealed during the initial interview. In the SZ group, the first-episode status was defined as having received the first psychiatric diagnosis less than one year prior to being engaged in the study, and by the presence of any form of psychotic symptoms for no longer than three years. SZ subjects underwent brain structural imaging (MRI) about a month before participation in order to exclude those with any acquired brain injuries (tumors, cerebrovascular changes, signs of TBI etc.). All patients were treated with stable doses of atypical antipsychotics during cognitive and electrophysiological assessment, which took place in the last week of hospitalization when a significant clinical improvement had been determined by treatment providing psychiatrists. The PANSS^65^ total score lower than 75 points was considered as confirming symptomatic remission, which concerned 85.71% of the finally compiled patients’ group. The premorbid IQ of all participants was estimated with Polish Adult Reading Test (PART) - a validated, standardized, and normalized Polish version of NART^66^. All participants provided informed consent for a protocol approved by the Bioethical Commission of the Medical University of Lublin, Poland, all methods were performed in accordance with relevant guidelines and regulations.

### Cognitive assessment

A digitized version of a processing speed test (Digit Symbols Test, DST) was applied to measure cognitive speed. A comprehensive description of this task has been reported elsewhere^67,68^. Generally, the task used in this study is based on Symbol Coding/Digit Symbol tests or similar methods included in various cognitive batteries, e.g., WAIS^69^ or BACS^70^. The task of the participant is to match numbers to abstract symbols as fast as possible. The idea to develop an original computerized version of the test in a form of a tablet application resulted from the necessity to provide access to all the raw data containing temporal parameters of task performance (in milliseconds). The raw data contain a set of reaction times (RTs) covering the intervals between displaying a given symbol and the indication of the corresponding digit. Further, we used this task in a digital form to reduce the potential impact of motoric disorders that might affect the results of paper-pencil tests, such as in Symbol Coding. In the applied version, participants had to only touch the appropriate stimulus. All subjects were tested with the same software and hardware (Lenovo Yoga 2 tablet with 13, 3″ screen, resolution Quad HD 2560 × 1440 IPS, Android™ 4.4 system).

Having all the reaction times from the cognitive speed test, it was possible to determine the intergroup difference regarding the extent of variability in information processing with the individual standard deviation (iSD) indicator. However, in clinical populations the distribution of RTs deviates from the Gaussian model^71,72^, therefore, to circumvent this limitation, the so-called ex-Gaussian modelling methods were applied, providing quantitative data regarding the properties of RTs distribution^73,74^, in a form of three independent parameters: (1) *mu* (*μ*) representing the mean of the normal component and reflecting average performance, (2) *sigma* (*σ*) being a symmetrical standard deviation of the normal component, and (3) *tau* (*τ*), the exponential part of the distribution with the most prolonged RTs. Typically *τ* was considered as an indicator of “attentional lapses” or “off-task behaviours” caused by a transient failure of goal maintenance^48,75,76^. Therefore, the analysis of the RTs distribution derived from tests of cognitive speed showed to what extent the SZ patients’ test outcome results from an elongation of average RTs, or from being off-task due to dysfunctions of executive control over the ongoing cognitive activity.

The modelling of ex-Gaussian parameters (*μ*, *σ*, *τ*) based the raw RTs derived from processing speed task has been conducted with the MATLAB toolbox “DISTRIB” (version: R2017a, Mathworks Inc. Natick, MA, USA). The data were pre-processed to guarantee the correct export of the results to the MATLAB software and proceeded with an individually customized Excel macro. All further operations were performed according to the recommendations of Lacouture and Cousineau^77^ for extracting the ex-Gaussian parameters from experimental data.

### Electroencephalographic recording

For all subjects, ten minutes of resting state (eyes closed) EEG data was recorded using a 21 scalp location Electro-cap (Electro-Cap International Inc., Ohio, USA) and Ag/AgCl disk electrodes. Electrodes have been deployed according to 10–20 International system (Fp1, Fp2, F3, F4, C3, C4, P3, P4, O1, O2, A1, A2, F7, F8, T3, T4, T5, T6, Fz, Pz, and Cz). The reference electrode was placed at the FCz. During the recordings, the impedance value of the electrodes was kept below 5 kΩ with the sampling rate of 500 Hz. The data were band-pass filtered from 0.5 to 70 Hz and, with an active notch filter, set at 50 Hz. After having been recorded, the data were exported to ASCII format. For further analysis, the ASCII files were imported to EEGLAB v.13.5.4b1^78^, an open source toolbox for MATLAB (Mathworks, Inc.). Then, each EEG recording was offline filtered with a bandpass Hamming window 0.5–45 Hz filter, and re-referenced offline to the common average reference. The common average reference was calculated with the exclusion of Fp1/2 and A1/2 electrodes because of contamination by EMG artifacts, electrodes A1/2 were excluded from further analysis. The data were then segmented into 75 epochs with the duration of 4096 samples (approximately 8 s) each. All of the epochs were visually inspected by a certified clinical neurophysiologist who removed from the analysis epochs contained by artifacts, such as head or muscle movements, jaw clinching and electrode cable movements. Finally, for each participant, thirty artifacts free epochs of 4096 samples were selected. As the number of the selected epochs across participants should be equal, and to avoid excluding additional subjects from the analysis, due to the number of artifacts in the recordings, we chose the number of 30 artifacts free epochs as the safe value for further analysis. In the next step, the epochs were band-pass filtered into the frequencies: delta (0.5–4 Hz), theta (4–8 Hz), lower alpha (8–10 Hz), upper alpha (10–12 Hz), beta (13–30 Hz) and low gamma (30–48 Hz). Due to the fact that different frequency ranges in an alpha band are involved in different cognitive processes^79^, the alpha band was divided into two frequency bands. The activity in gamma frequency was also assessed due to its crucial role in electroencephalographic studies in schizophrenia^80,81^. For the functional connectivity and MST analysis, all epochs in ASCII format were exported to Brainwave 0.9.152.4.1 (available free at http://home.kpn.nl/stam7883/brainwave.html). The EEG recordings took place around noon, between 10 am and 4 pm, in a quiet, well-lit room. Smokers were asked to refrain from smoking at least an hour before the neurophysiological examination.

### Functional connectivity

To assess the functional connectivity strength for each frequency epoch separately and also for all possible pairs of electrodes, the phase lag index (PLI) has been applied^82^. PLI provides a measure of phase synchronization based on the asymmetry of the distribution of instantaneous phase differences between two time series. The instantaneous phase differences were computed using the analytical signal concept and the Hilbert transform. Owing to the fact that zero-lag synchronization has been removed from the analysis, the PLI outcome is less affected by the influence of common source in comparison to i.e. synchronization likelihood or phase coherence.

### Reconstruction of the neural networks: a Minimum Spanning Tree

The minimum spanning tree is built on the basis of Kruskal’s algorithm. It starts by ordering the weight of all edges and then the stronger edges, ie. with highest PLI, became connected, however, excluding those connections that form loops. These steps are performed as many times as possible and at the end, the final tree contains 19 nodes and 18 edges, considering the number of all electrodes used in our EEG recording. The MST metrics were calculated based on all PLI matrix, in every frequency band, separately. The MST enables computation of many parameters, however, they are strongly correlated and, therefore, redundant^83^. The limitation of the number of MST metrics included in statistical analysis was also guided by an attempt to avoid type 1 error. According to van Diessen *et al*. (2016), MST diameter and leaf number or leaf fraction, are two most straightforward global metrics informative of the MST network topology. Moreover, they are sufficient to classify the networks as more line-like, in which case the diameter increases and the leaf number decreases, or star-like, with high leaf number and low diameter^27^. A degree parameter is defined as the number of links connected to a node, calculated for each node separately. In this study a maximal degree value was computed to characterize the degree of the whole tree. A leaf fraction refers to the number of nodes on the tree with degree = 1, it can be defined as L_f_ = L/m, where L is the leaf number. The leaf number ranges from 2 (typical of a line-like topology) and a maximum value m = N-1 (a star-like topology). The leaf number is associated with the tree diameter (d), defined as the largest distance between any two nodes. The upper limit of the diameter is defined as d_max_ = m-L + 2, so, the largest possible diameter decreases when the leaf number increases. To compare the groups with regard to network hub measure, the betweenness centrality (BC) was also computed, defined as the number of the shortest paths between any pair of nodes *i* and *j* in the network passing through a node *u*, divided by the total number of paths between *i* and *j*. BC values range from 0 for the leaf node and 1 for the central node in a star-like network where it has the highest load. Additional parameter has been provided, a maximal betweenness centrality (BC_max_), an indicator of the hub’s strength, which is representative for the node(s) with the highest BC in the tree^29^.

Because the previously discussed research indicates that the network of SZ patients is more randomly organized than in the case of healthy individuals^84^, a set of MST metrics computed in this study included a degree correlation (R), estimating how strong the degree of a node is dependent on the degree of its neighbouring vertices to which it is connected. A positive value of degree correlation indicates that the graph is assortative and a negative value means that the graph is disassortative^29,30,50^.

The regional betweenness centrality was computed separately, as averaged BC for nodes assigned as belonging to anterior (nodes: F_p_1, F_p_2, F_7_, F_8_, F_3_, F_4_, F_z_) and posterior areas (nodes: T_5_, T_6_, P_3_, P_4_, P_z_, O_1_, O_2_). Exactly the same electrodes assigned as covering anterior or posterior regions were chosen in the study of Symond *et al*.^85^ of task-related gamma synchrony in first-episode schizophrenia. The central region (C_3_, C_4_, C_z_) was not included in the statistical analysis because the aim of the study was to identify the nodes/hubs as unambiguously frontal or non-frontal. The inclusion of an equal number of nodes in two areas should also reduce any bias in computing a regional centrality.

### Data analysis and statistics

To compare the groups in terms of demographical and clinical variables χ^2^ test and one-way ANOVA were applied with partial eta squared (η_p_^2^) as an effect size indicator. The between-group differences in terms of cognitive variables (the sum of correctly matched stimuli, mean RT, iSD and the number of errors committed in the processing speed task), including ex-Gaussian parameters (*μ*, *σ*, *τ*) were tested with ANCOVA, including age, education and premorbid IQ as controlled covariates. In this case, all between-group comparisons performed with controlled covariates were carried out individually for each dependent cognitive variable. The Bonferroni correction was applied to establish statistical significance threshold resulting from multiple testing of controlled covariates. After determining the distributional parameters significantly differentiating the groups, correlations between these parameters were calculated separately for SZ and HC groups to verify the potential dependencies between ex-Gaussian indicators. The next step was to perform regression analysis with the overall result in the DST test as a dependent variable and ex-Gaussian parameters, which differed the groups as independent variables to determine which of them could account for cognitive slowdown in the SZ group. In the SZ group, associations between the mentioned ex-Gaussian parameters and clinical variables were also identified with application of two-tailed Spearman correlation (R). Next, raw PLI and MST results averaged across the epochs per subject were log-transformed [y = ln(x)] to ensure distributional normality of these variables. To avoid zeros in transformed data, they were computed with additional 1 · 10^−24^ correction. Group differences regarding individual PLI and MST (diameter, leaf fraction, degree correlation, BC_max_, and global BC as whole-brain network metrics, and anterior and posterior BC as regional metrics) results were tested with ANCOVA, again, with age, education and premorbid IQ as controlled covariates and η_p_^2^ as a measure of effect size. Similarly as in the case of cognitive variables comparisons, the Bonferroni correction was used to set a statistical significance threshold. After establishing the functional connectivity and network indicators differentiating the studied groups, they were correlated with the ex-Gaussian parameters being significant predictors of cognitive speed in the SZ sample. In this case, the Pearson’s r correlation coefficient was applied. To control for the hypothetical impact of clinical independent variables on cognitive–network relationships in the SZ group, a multivariate regression analysis was performed with ex-Gaussian parameters as dependent variables and MST results as independent variables, including the duration of illness, duration of untreated psychosis, and the risperidone equivalent as controlled predictors. Only the variables which significantly differentiated the studied groups and uncorrelated predictors were included. Additionally, the potential correlations between MST parameters and psychopathology assessed with the PANSS scale were verified. Due to the fact that the PANSS results deviated from the normal distribution, the two-tailed Spearman R coefficient was used. For all correlations coefficients, p values of less than 0.01 were regarded as statistically significant. Establishing this threshold for correlation resulted from an attempt to avoid false positive effects, which could have been a consequence of potential redundancy or similarity of the analyzed MST parameters.

## Supplementary information

Supplementary materials
