# Supplementary material for: Abnormalities in hubs location and nodes centrality predict cognitive slowing and increased performance variability in first-episode schizophrenia patients
Source: Sci Rep. 2019 Jul 3;9:9594. doi: 10.1038/s41598-019-46111-0 (PMC6610093; doi:10.1038/s41598-019-46111-0)
Supplement: Supplementary file 1 — Supplementary materials [file 41598_2019_46111_MOESM1_ESM.docx]

SUPPLEMENTARY INFORMATION

*Abnormalities in hubs location and nodes centrality predict cognitive slowing*

*and increased performance variability in first-episode schizophrenia patients*

Paweł Krukow, Kamil Jonak, Robert Karpiński, Hanna Karakuła-Juchnowicz

Figures S1. and S2. presented below contain the minimal spanning tree graphs, with channel numbers changed to electrodes markings according to international 10 – 20 system, being an output from the Brainwave 0.9.152.4.1 software (available free at <http://home.kpn.nl/stam7883/brainwave.html>) for patients with schizophrenia (SZ) and healthy controls (HC), computed separately for beta and gamma frequencies. Graphs were obtained by averaging PLI adjacency matrix in a given frequency for a whole group, and then computing the MST matrix and generating final raw minimal spanning trees, as it was presented in Figure 1. containing the neurophysiological data analysis pipeline. Graphs for beta and gamma bands were chosen because these frequencies contained the largest number of MST metrics statistically differentiating the studied groups, in addition, the MST indicators in these bands turned out to be significant correlates or predictors of psychopathological symptoms and cognitive deficits assessed in the first-episode schizophrenia group.

HC SZ


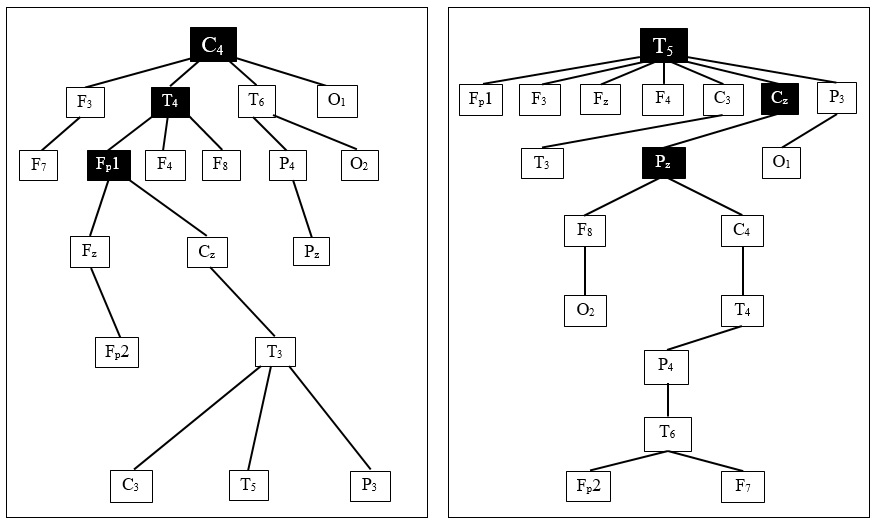


Figure S1. Minimum spanning trees for both groups in beta frequency. Nodes with black filling are those with the highest betweenness centrality in the tree.


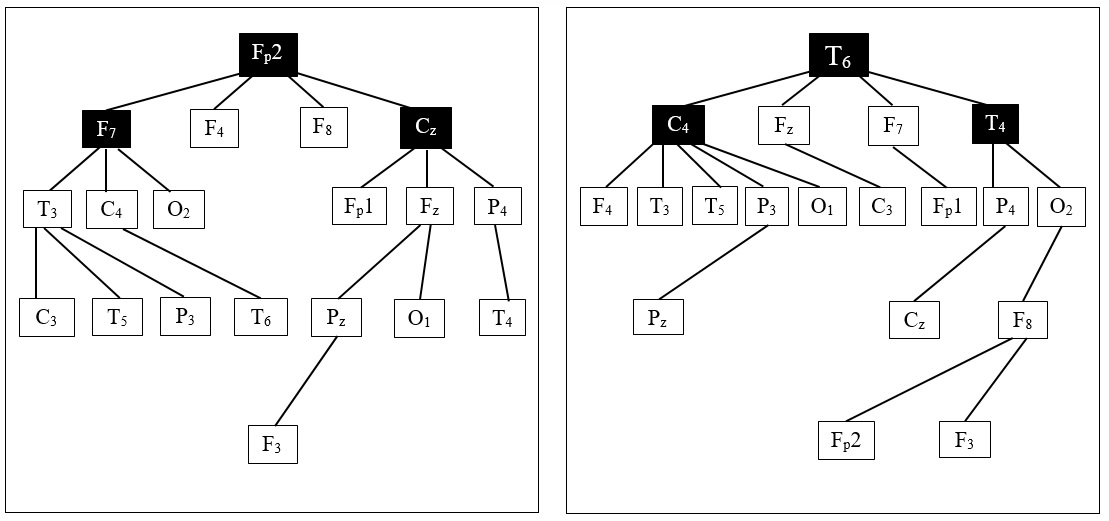


Figure S2. Minimum spanning trees for both groups in gamma frequency. Nodes with black filling are those with the highest betweenness centrality in the tree.
